# Sequential intravenous allogeneic mesenchymal stromal cells as a potential treatment for thromboangiitis obliterans (Buerger’s disease)

**DOI:** 10.1186/s13287-018-0901-6

**Published:** 2018-05-30

**Authors:** Jorge D. Martin-Rufino, Francisco S. Lozano, Alba M. Redondo, Eva M. Villaron, Raquel Rueda, Rafael Fernandez-Samos, Fermin Sanchez-Guijo

**Affiliations:** 1 Department of Hematology, Cell Therapy Unit, IBSAL-Hospital Universitario de Salamanca, Paseo de San Vicente 58-182, 37007 Salamanca, Spain; 2 Department of Angiology and Vascular Surgery, IBSAL-Hospital Universitario de Salamanca, Salamanca, Spain; 3 Faculty of Medicine, Universidad de Salamanca, Salamanca, Spain; 4 Department of Radiology, Hospital de Leon, Leon, Spain; 5 Department of Angiology and Vascular Surgery, Hospital de Leon, Leon, Spain; 6 Centro en Red de Medicina Regenerativa y Terapia Celular de Castilla y Leon, Salamanca, Spain

**Keywords:** Allogeneic mesenchymal stromal cells, Thromboangiitis obliterans, Cell transplantation

## Abstract

Thromboangiitis obliterans (TAO), also known as Buerger’s Disease, is an occlusive vasculitis linked with high morbidity and amputation risk. To date, TAO is deemed incurable due to the lack of a definitive treatment. The immune system and inflammation are proposed to play a central role in TAO pathogenesis. Due to their immunomodulatory effects, mesenchymal stromal cells (MSCs) are the subject of intense research for the treatment of a wide range of immune-mediated diseases. Thus far, local intramuscular injections of autologous or allogeneic MSCs have shown promising results in TAO. However, sequential intravenous allogeneic MSC administration has not yet been explored, which we hypothesized could exert a systemic anti-inflammatory effect in the vasculature and modulate the immune response. Here, we report the first case of a TAO patient at amputation risk treated with four sequential intravenous infusions of bone marrow-derived allogeneic MSCs from a healthy donor. Following administration, there was significant regression of foot skin ulcers and improvements in rest pain, Walking Impairment Questionnaire scores, and quality of life. Sixteen months after the infusion, the patient had not required any further amputations. This report highlights the potential of sequential allogeneic MSC infusions as an effective treatment for TAO, warranting further studies to compare this approach with the more conventionally used intramuscular MSC administration and other cell-based therapies.

## Introduction

Thromboangiitis obliterans (TAO), also known as Buerger’s disease, is an inflammatory occlusive disorder that affects small and medium sized peripheral blood vessels of the extremities. It is characterized by hypercellular inflammatory thrombotic occlusions of arteries and veins, which ultimately leads to vascular insufficiency, critical limb ischemia, and amputation [1]. This high-morbidity disease mainly affects young male smokers, severely limiting their quality of life. Although smoking cessation is the most effective therapeutic intervention, there is currently no definitive cure for TAO [2].

To date, the pathogenesis of TAO has not been fully elucidated. Smoking is considered the main precipitating factor of the disease which could trigger an immune response and inflammatory damage targeting vascular endothelial cells and leading to thrombosis [3]. Indeed, several reports have provided insights into the immunopathogenesis of TAO, suggesting that the immune system plays a critical role in the etiology of the disease [1, 3–5].

Mesenchymal stromal cells (MSCs) are the subject of intense research over a wide range of conditions due to their angiogenic and immunomodulatory effects [6]. Previous studies using MSCs for TAO have focused on their local effect after intramuscular administration [7, 8]. However, we hypothesized that their intravenous use could directly act upon the mechanisms that underlie TAO pathogenesis by exerting systemic anti-inflammatory effects in the vasculature and modulating the response of the immune system. Sequential doses of intravenous MSCs have been previously shown to be safe and potentially effective in the treatment of cardiovascular conditions and immune complications, such as graft-versus-host disease (GVHD), through systemic immunomodulatory mechanisms [9, 10]. In addition, the use of allogeneic MSCs could overcome the problems of autologous MSCs in inflammatory diseases in which they are dysfunctional [11].

To the best of our knowledge, this is the first report of a TAO patient treated with sequential intravenous infusions of allogeneic MSCs. The patient, who had critical limb ischemia and was at amputation risk, had exhausted all available therapeutic options and received intravenous allogeneic MSCs under a compassionate use program.

## Methods

### Patient and pretreatment assessment

A 41-year-old man, diagnosed with TAO and suffering from critical chronic ischemia and ulcerous lesions on his right lower leg, was referred to the Angiology and Vascular Surgery Department to assess his eligibility for treatment with MSCs under a compassionate use program. He had developed ulcers and critical ischemia on the left lower leg, despite smoking cessation, 8 years before. A left lumbar sympatectomy and the implantation of an epidural spinal cord neurostimulator had been performed, but a left transtibial amputation was necessary 4 years before after an unsuccessful femoropopliteal bypass.

**Fig. 1:**
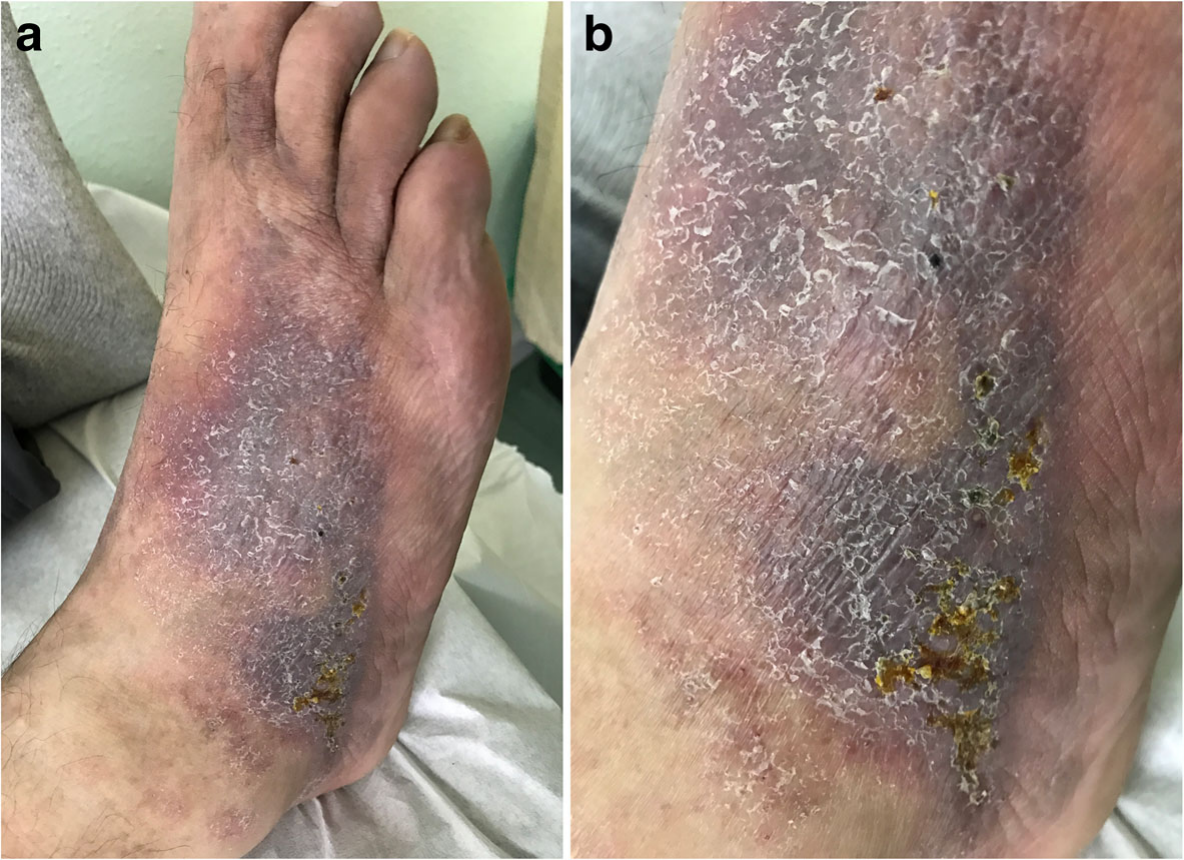
The patient’s right foot before MSC treatment. Prior to intravenous allogeneic MSC sequential infusions, trophic changes and multiple punctate ulcers were visible in the patient’s right foot (**a**). Close-up view of the right foot dorsum (**b**)

During our initial consultation, the patient complained of severe rest pain and paresthetic symptoms in his right lower limb. The extremity displayed pallor and coolness and the pedal pulse was absent on examination. Remarkably, all the dorsum of the right foot showed trophic changes with multiple punctate ulcers (Fig. 1a, b). The ankle-brachial index (ABI) was 0.66. The patient’s treatment included clopidogrel, pentoxifylline, amlodipine, and buprenorphine transdermal patches.

The Walking Impairment Questionnaire (WIQ) was used to quantitatively assess the impact of MSC treatment on the patient’s walking capability. Each WIQ metric is scored from 0 (total incapacity) to 100 (full capacity) [12]. The patient’s WIQ distance score was 54, the speed score 31, and the climbing score 67.

The European Quality of Life—5 dimensions (EQ-5D) questionnaire was used to assess changes in the patient’s health-related quality of life. The patient’s EQ-5D descriptive system score was 0.72 out of 1, which analyzes mobility, self-care, usual activities, pain/discomfort, and anxiety/depression. The EQ-5D visual analogue scale (VAS) score was 70, with 0 and 100 being the ‘worst’ and the ‘best’ imaginable health states, respectively [13].

**Fig. 2:**
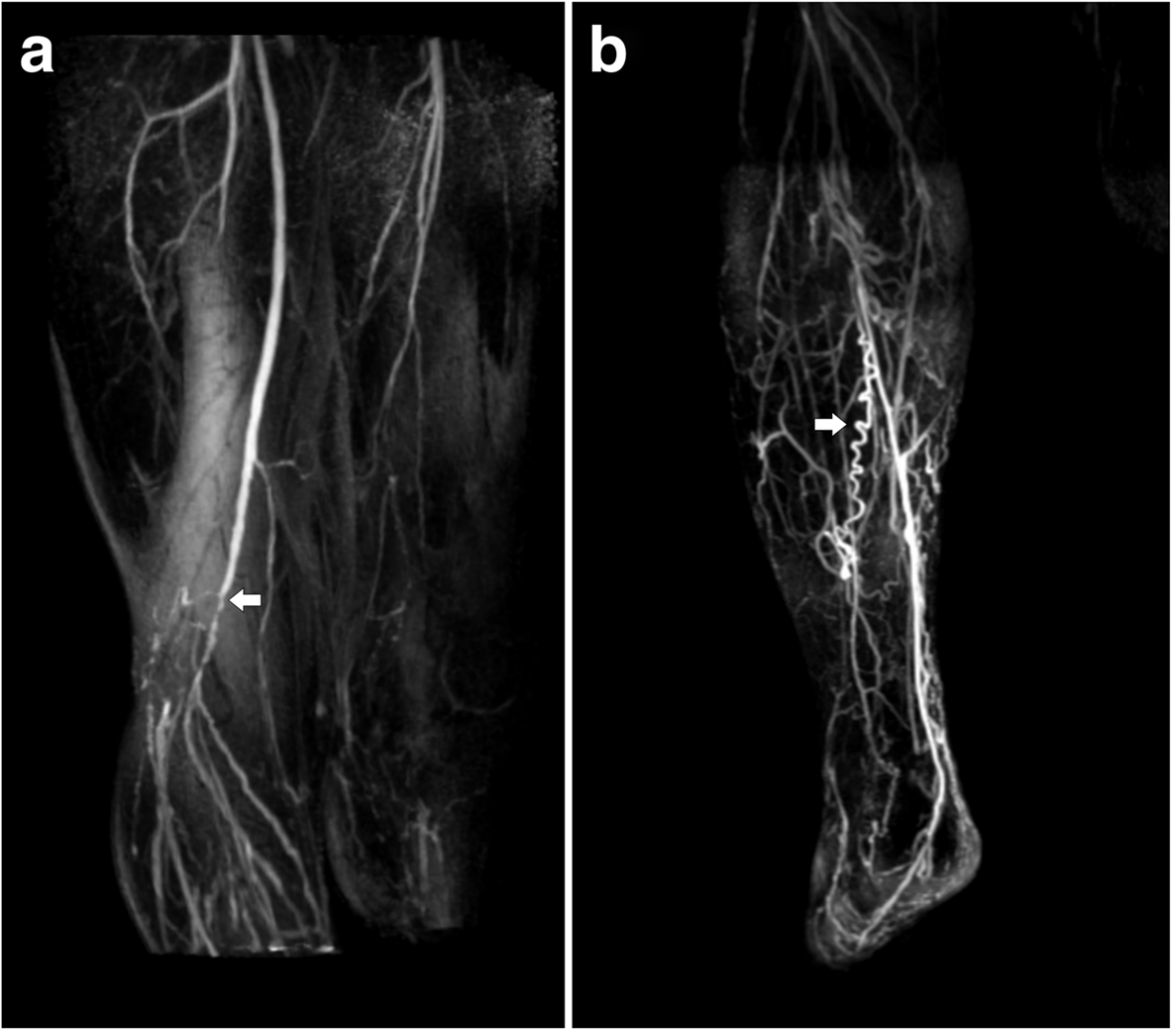
MRA of the patient’s right lower limb. Subtracted maximum intensity projection (MIP) contrast-enhanced MRA of the thighs (**a**) and the right calf (**b**). Arrows indicate multiple critical stenoses in the right limb in **a** and characteristic corkscrew-like collateral vessels in **b**

Magnetic resonance angiography (MRA) studies revealed a right patent femoral artery until the origin of the popliteal artery, which displayed multiple critical stenoses and a complete occlusion. Abundant collateral circulation and tortuous corkscrew-like vessels, characteristic features of the disease, were also present. The right posterior tibial artery was the only clearly identifiable distal branch (Fig. 2a, b).

In the patient’s situation, no therapeutic alternatives were available to stop the progression of trophic changes or irrepressible pain, so ongoing clinical worsening could have soon required a new amputation. Therefore, due to the rationale indicated above, sequential intravenous infusions of allogeneic MSCs were planned.

### MSC production

All the procedures that follow were in accordance with the ethical standards of the Helsinki Declaration and were approved by the Ethics Committee of the Hospital Universitario de Salamanca. Informed consent was obtained from the MSC-treated TAO patient and the MSC donor. MSCs were expanded following Good Manufacturing Practice guidelines in the Spanish Medicines Agency-accredited Cell Production Unit of the Hospital Universitario de Salamanca, as previously described [10].

Briefly, following standard operating procedures, 85 mL of bone marrow were harvested from a healthy 42-year-old female donor. A total of 3.04 × 10^8^ mononuclear cells were isolated by Ficoll-Paque density-gradient centrifugation (GE Healthcare BioSciences, AB, Uppsala, Sweden). Following resuspension, mononuclear cells were plated in noncoated polystyrene flasks (Corning Costar, Celbio, Milan, Italy) in modified Eagle’s medium-α, supplemented with 1% penicillin/streptomycin (Pen/Strep; Gibco, Paisley, UK) and 5% platelet lysate. MSCs were expanded at 37 °C, 5% CO_2_, and 90% relative humidity. The medium was changed twice a week.

Human platelet lysate was obtained by pooling 4 or 5 platelet units (300–400 mL/unit) after a freeze/thaw cycle (−80 °C to 37 °C). Platelets were used irrespective of their ABO type. Samples underwent centrifugation at 900 g for 30 min; the supernatants were used as a supplement and heparin (2 IU/mL of medium) was added to prevent gel formation.

After expansion, a total dose of 3.40 × 10^8^ MSCs was frozen in four cryopreservation bags using a rate-controlled freezing device (CM-2010 Biological Freezer, Carburos Medica, Madrid, Spain). Cells were cryopreserved in 10% dimethyl sulfoxide (DMSO) and 90% human AB plasma solution and stored in liquid nitrogen until thawing.

### MSC characterization

MSCs were characterized by flow cytometric analysis of MSC-associated cell surface markers and multilineage differentiation assays, as previously described [10]. Additionally, MSCs were karyotyped following our previously reported optimized procedure [14].

MSC-associated cell surface marker analysis was performed by incubating 200,000 MSCs with a combination of antibodies: CD90-fluorescein isothiocyanate (FITC), CD73-phycoerythrin (PE), CD45-peridinin chlorophyll protein (PerCP), CD34-FITC, CD19-PerCP, CD166-PE, HLA-DR PerCP.Cy5.5, CD14-PE (all manufactured by Becton-Dickinson BD, San Diego, CA), CD44-FICT (Immunostep, Salamanca, Spain), and CD105-allophycocyanin (R&D Systems, Minneapolis, MN). Cells were analyzed using a FACSCalibur cytometer (BD Biosciences, San Jose, CA) and the Infinicyt Software (Cytognos, Salamanca, Spain).

MSC phenotype was confirmed by successfully inducing differentiation into adipogenic, chondrogenic, and osteogenic mesenchymal lineages. All multilineage differentiation assays were conducted in Miltenyi Biotec differentiation media (Bergisch Gladbach, Germany). To verify osteogenic differentiation, alkaline phosphatase activity was measured after NBT/BCIP staining (nitroblue tetrazolium chloride/5-bromo-4-chloro-3-indolyl-phosphate) (Roche, Basel, Switzerland). Adipogenic differentiation was verified by Oil Red O staining (Certistain Merck KGaA, Darmstadt, Germany). Finally, chondrogenic differentiation was verified by immunostaining using a collagen type II-specific mouse anti-human monoclonal antibody (Chemicon International, Hofheim, Germany). Release testing before administration comprised morphology (fibroblast-like adherent cells), viability (> 70% live cells measured by Trypan Blue assay), purity by FACS (> 70% CD90^+^, CD44^+^, CD73^+^, CD166^+^, CD34^−^, CD45^−^, CD19^−^, CD14^−^, and HLA-DR^−^ cells), normal karyotype, osteogenic and adipogenic differentiation assays, and mycoplasma and sterility testing. Cell viability was 100% before freezing and > 90% after thawing in all infusions.

### Infusion

Four intravenous MSC infusions were performed in total, on days 1, 4, 11, and 18. Each dose contained 8.5 × 10^7^ cells (1.06 × 10^6^ cells/kg). To prevent DMSO-derived toxicity, intravenous premedication with 100 mg hydrocortisone sodium phosphate, 10 mg dexchlorpheniramine, and 1 g paracetamol was administered, and vital signs were monitored after infusion. The scheme employed followed the PEI-06-076 Investigational Medicinal Product Dossier (IMPD), approved by the Spanish Medicines Agency (AEMPS) to produce allogeneic MSCs for immune-based diseases, as previously published [10].

## Results

No adverse effects or signs of allograft rejection were detected following any of the four intravenous allogeneic MSC infusions.

**Fig. 3:**
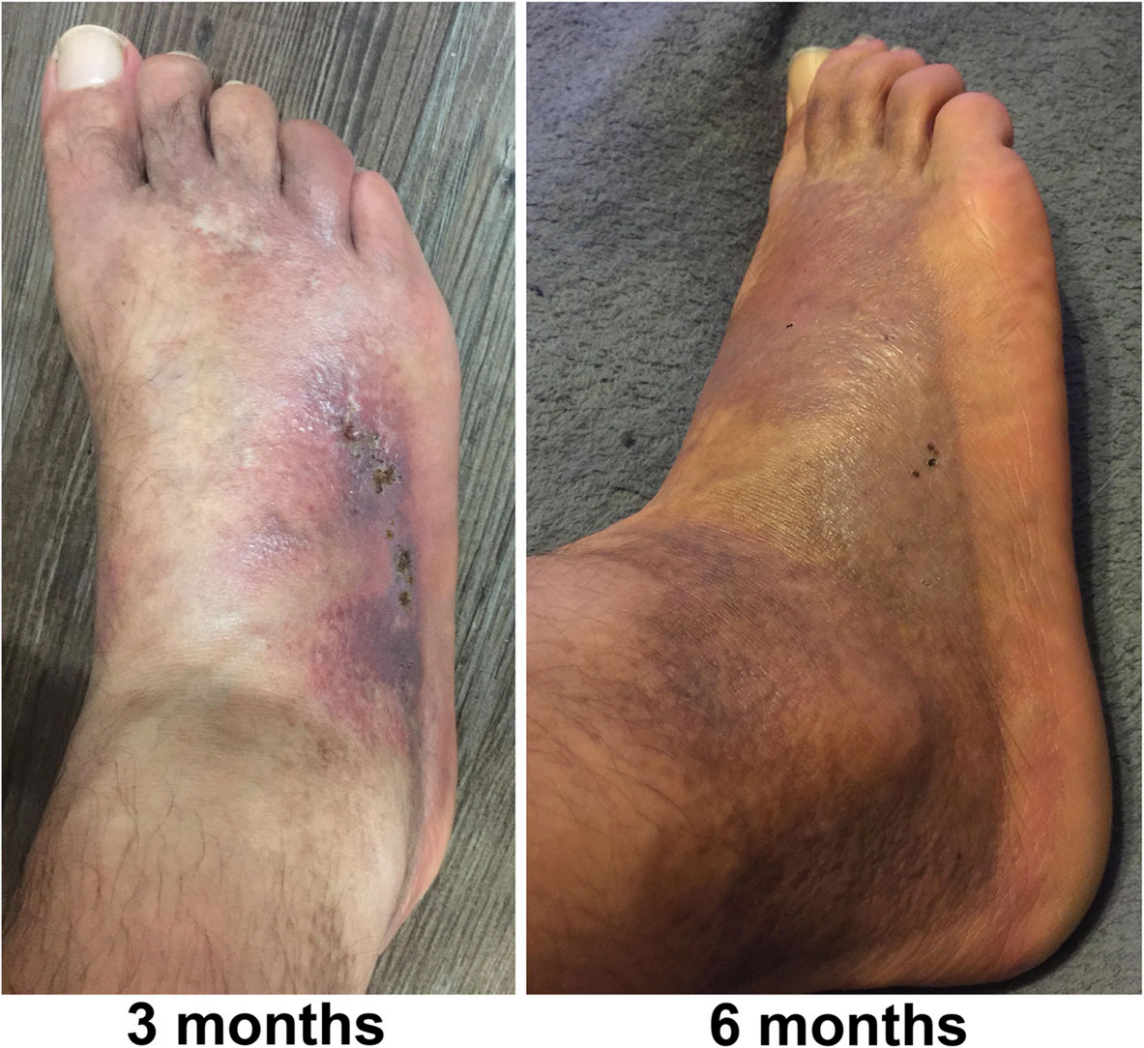
The patient’s right foot after four sequential intravenous infusions of allogeneic MSCs. A significant regression of trophic changes was observed 3 months after the last infusion. Near complete remission of skin lesions and ulcers was seen 6 months after the treatment

Three months after the treatment, a regression of trophic changes in the patient’s right foot could be observed (Fig. 3). Six months after the infusions, the patient had almost complete ulcer remission and restitution of the skin integrity (Fig. 3).

In the 5-month follow-up examination, the patient reported a reduction in rest pain and the disappearance of paresthesia. The pedal pulse was again palpable and an ABI of 0.47 was measured. However, ABI correlation with functional performance is controversial [15].

**Fig. 4:**
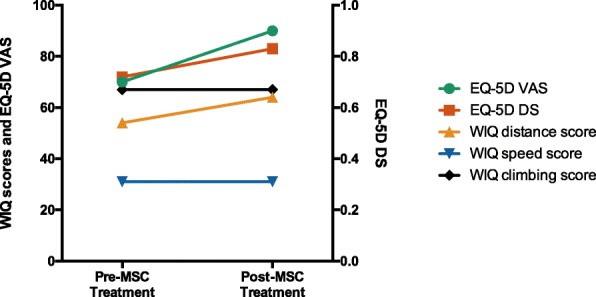
Walking Impairment Questionnaire (WIQ) and European Quality of Life–5 dimensions (EQ-5D) scores before and 5 months after intravenous allogeneic mesenchymal stromal cell (MSC) infusions. DS descriptive system, VAS visual analogue scale

The patient’s WIQ distance score increased from 54 to 64 out of 100, and the speed and climbing scores remained unaltered. Furthermore, the EQ-5D scores improved from 0.72 to 0.83 out of 1 in the descriptive system and to 90 out of 100 in the VAS, revealing a marked quality of life improvement (Fig. 4). An MRA performed 5 months after the first infusion showed no significant changes in the vasculature.

Ten months after finishing the treatment, a new spinal cord neurostimulator was implanted to improve pain management. Sixteen months after the MSC infusions, the patient had not required any further major nor minor amputations.

Since the donor and the recipient only shared two out of six HLA antigens (one HLA-A and one HLA-DR antigen by conventional serological studies), the patient’s serum was screened for the presence of anti-HLA antibodies (IgG isotype) by Luminex® single antigen determination. No anti-HLA antibodies were detected.

## Discussion

The patient was referred to our hospital in the same clinical situation that resulted in the amputation of his left leg several years before, with no therapeutic options left in the event of clinical worsening. After the joint assessment of the patient between the Vascular Surgery Department and the Cell Therapy Unit, which had collaborated in the past for the treatment of critical limb ischemia patients, the administration of MSCs under compassionate use was decided. In this report, the quality of life and clinical improvement suggest that sequential intravenous infusions of allogeneic MSCs might be an effective treatment for TAO. In the future, clinical trials with larger patient numbers are needed to demonstrate safety and efficacy, which would also be an opportunity to perform additional studies to assess the immune-system changes induced by MSCs in this clinical entity.

The therapeutic approach in this patient sought a systemic immunomodulatory effect on the vasculature and the immune system, rather than a local one, through the sequential intravenous infusions of allogeneic MSCs. A central role in the pathogenesis of TAO has been attributed to the immune system, based on the existence of immunocompetent cells in acute lesions and the identification of elevated pro- and anti-inflammatory cytokines and autoantibodies in patient sera [1]. Recently, it has been proposed that smoking, the main risk factor of the disease, could induce IL-33-mediated immune responses that would result in vascular endothelial damage with subsequent thrombosis and ischemia [3]. Therefore, sequential intravenous infusions of allogeneic MSCs could potentially target the mechanisms underlying TAO immunopathogenesis.

To the best of our knowledge, this is the first description of a TAO patient treated with sequential intravenous infusions of allogeneic MSCs. A recent report has demonstrated that, when injected intravenously, MSCs become trapped in the lungs and phagocytized by cells of the innate immune system. These cells rapidly distribute the MSC effect to distant body organs after polarization towards an immunoregulatory phenotype [16]. The immunomodulatory properties of MSCs after intravenous infusion have been highlighted in numerous disease models and clinical studies, and have shown enormous potential in the treatment of immune-mediated diseases, such as GVHD [6, 17, 18].

With hundreds of completed or ongoing clinical trials [19], allogeneic MSCs are considered safe and may be superior to autologous MSCs that are dysfunctional in inflammatory diseases [11]. Furthermore, this report emphasizes the advantages of the intravenous route as a convenient method for the administration of multiple MSC doses in the outpatient setting.

## Conclusion

This report shows that sequential intravenous administration of allogeneic MSCs might be an effective treatment for TAO. Our results warrant further studies to compare this approach with the more conventionally used intramuscular MSC administration and with other cell-based therapies in order to determine the most effective approach for TAO in larger clinical trials.

## Abbreviations

ABI: Ankle-brachial index
EQ-5D: European Quality of Life—5 dimensions
GVHD: Graft-versus-host disease
MRA: Magnetic resonance angiography
MSC: Mesenchymal stromal cell
TAO: Thromboangiitis obliterans
VAS: Visual analogue scale
WIQ: Walking Impairment Questionnaire

## References

[1] Klein-Weigel PF, Richter JG (2014). Thromboangiitis obliterans (Buerger’s disease). Vasa.

[2] Rivera-Chavarría IJ, Brenes-Gutiérrez JD (2016). Thromboangiitis obliterans (Buerger’s disease). Ann Med Surg.

[3] Sun XL, Law BY, de Seabra Rodrigues Dias IR, Mok SWF, He YZ, Wong VK. Pathogenesis of thromboangiitis obliterans: gene polymorphism and immunoregulation of human vascular endothelial cells. Atherosclerosis. 2017;265:258–65. 10.1016/j.atherosclerosis.2017.08.009 28864202

[4] Dellalibera-Joviliano R, Joviliano EE, Silva JS, Evora PRB (2012). Activation of cytokines corroborate with development of inflammation and autoimmunity in thromboangiitis obliterans patients. Clin Exp Immunol.

[5] Ketha SS, Cooper LT (2013). The role of autoimmunity in thromboangiitis obliterans (Buerger’s disease). Ann N Y Acad Sci.

[6] Gao F, Chiu SM, Motan DAL, Zhang Z, Chen L, Ji H-L (2016). Mesenchymal stem cells and immunomodulation: current status and future prospects. Cell Death Dis.

[7] Gupta PK, Krishna M, Chullikana A, Desai S, Murugesan R, Dutta S (2017). Administration of adult human bone marrow-derived, cultured, pooled, allogeneic mesenchymal stromal cells in critical limb ischemia due to Buerger’s disease: phase ii study report suggests clinical efficacy. Stem Cells Transl Med.

[8] Ra JC, Jeong EC, Kang SK, Lee SJ, Choi KH (2017). A prospective, nonrandomized, no placebo-controlled, phase I/II clinical trial assessing the safety and efficacy of intramuscular injection of autologous adipose tissue-derived mesenchymal stem cells in patients with severe Buerger’s disease. Cell Med.

[9] Luger D, Lipinski MJ, Westman PC, Glover DK, Dimastromatteo J, Frias JC (2017). Intravenously-delivered mesenchymal stem cells: systemic anti-inflammatory effects improve left ventricular dysfunction in acute myocardial infarction and ischemic cardiomyopathy. Circ Res.

[10] Sánchez-Guijo F, Caballero-Velázquez T, López-Villar O, Redondo A, Parody R, Martínez C (2014). Sequential third-party mesenchymal stromal cell therapy for refractory acute graft-versus-host disease. Biol Blood Marrow Transplant.

[11] Wang J, Liao L, Wang S, Tan J (2013). Cell therapy with autologous mesenchymal stem cells—how the disease process impacts clinical considerations. Cytotherapy.

[12] Nicolaï SPA, Kruidenier LM, Rouwet EV, Graffius K, Prins MH, Teijink JAW (2009). The walking impairment questionnaire: an effective tool to assess the effect of treatment in patients with intermittent claudication. J Vasc Surg.

[13] Rabin R, De Charro F (2001). EQ-5D: a measure of health status from the EuroQol Group. Ann Med.

[14] Muntión S, Sánchez-Guijo FM, Carrancio S, Villarón E, López O, Diez-Campelo M (2012). Optimisation of mesenchymal stromal cells karyotyping analysis: implications for clinical use. Transfus Med.

[15] Coutinho T, Rooke TW, Kullo IJ. Arterial dysfunction and functional performance in patients with peripheral artery disease: a review. Vasc Med. 2011;16:203–11. 10.1177/1358863X11400935 21447607

[16] de Witte SFH, Luk F, Sierra Parraga JM, Gargesha M, Merino A, Korevaar SS, et al. Immunomodulation by therapeutic mesenchymal stromal cells (MSC) is triggered through phagocytosis of MSC by monocytic cells. Stem Cells. 2018;36:602–15. 10.1002/stem.2779 29341339

[17] Amorin B, Alegretti AP, Valim V, Pezzi A, Laureano AM, da Silva MAL (2014). Mesenchymal stem cell therapy and acute graft-versus-host disease: a review. Hum Cell.

[18] Zhao Q, Ren H, Han Z (2016). Mesenchymal stem cells: immunomodulatory capability and clinical potential in immune diseases. J Cell Immunother.

[19] Galderisi U, Squillaro T, Peluso G (2016). Clinical trials with mesenchymal stem cells: an update. Cell Transplant.

